# Process Evaluation of a School-Based Program Aimed at Preventing Obesity in Adolescents from Lima and Callao, Peru

**DOI:** 10.3390/ijerph17134804

**Published:** 2020-07-03

**Authors:** Rosemary Cosme Chavez, Eun Woo Nam

**Affiliations:** 1 Yonsei Global Health Center, Yonsei University, Wonju 26493, Korea; cosmechavezrosemary@yonsei.ac.kr; 2 Department of Health Administration, Graduate School, Yonsei University, Wonju 26493, Korea

**Keywords:** process evaluation, school-based intervention, obesity, Peru, KOICA

## Abstract

The study aims to describe process evaluation measures of the three-year Health Promoting Schools’ obesity prevention program in Lima and Callao, Peru, and to assess factors that influenced the implementation of the intervention leading to the mentioned process outcomes results. The program was implemented in four public high schools located in low-income areas of Lima and Callao. Embedded in a Health Promoting School Intervention, the program consisted of two main components—an education program and several environmental activities. Quantitative data were collected and analyzed based on dose delivered and reach for each specific activity. Dose received was analyzed by satisfaction scores related to six specific activities. Furthermore, qualitative data including documentation of activities and transcriptions from individual in-depth interviews were qualitatively analyzed to identify factors influencing the implementation. The education component of the Health Promoting Schools’ program achieved a 78.4% average nutrition sessions delivery in 2015 and 88.0% in 2017; while for PA sessions, the average delivery in 2015 was of 79.7% and 93.8% in 2017. In the case of reach, at least 75% of total students participated in all sessions per year. Nevertheless, there were differences in terms of delivery and participation in a number of environmental activities within and between schools during the program period. Differences in delivery included education sessions for parents, teachers, Junior Health Promoters, and school food kiosk staff, besides execution of physical activity events every year. Impeding factors included the complexity of the overall intervention, limited strategies to facilitate program implementation and those to maintain the participation of subjects, and related contextual factors.

## 1. Introduction

While more emphasis has been placed on outcome evaluation to determine whether a health program has been successful or not, process evaluation, which allows us to understand and interpret such results, is equally important [1]. Process evaluation identifies the conditions for, or determinants of successful programs by trying to document what happened during the implementation of an intervention that could affect its impacts or outcomes [2]. A program’s failure may be related to an array of factors ranging from poor program design, variations in contents and style of delivery of intervention activities to failure in reaching the desired number of participants from the target group [3].

Over the last twenty years, literature on how to measure program implementation has become widely available. The increase in the use of process evaluation is partly related to the inherent characteristics of public health interventions, the complexity that results when interventions are implemented at multiple levels and with multiple audiences, and the accountability demands of results as in the case of Official Development Assistance (ODA)-funded programs [3,4]. While several models to guide the development of process evaluation have been developed, most have identified that factors related to program characteristics, implementers and context affect the outcomes obtained in public health programs [1,5,6,7]. However, not all of these influences have been extensively reported.

A commonly used framework is the one outlined by Steckler and Linnan (2002), which includes seven recommended elements of a process evaluation plan: Context (surrounding social systems such as structures and cultures of organizations and groups involved, inter-organizational linkages, existing legislation, and other concurrent events), reach (degree to which the intended target group participates in intervention components), dose delivered and received (amount of program delivered by implementers and extent to which participants responded to it), fidelity (extent to which an intervention is delivered as designed), implementation (a composite score of reach, dose, and fidelity) and, recruitment (procedures that were used to attract potential program participants and maintain their involvement) [2]. While there is evidence of different type of interventions using the aforementioned framework as a basis for their process evaluations, different studies argued that other aspects, such as the complexity of the program itself and the extent strategies are put in place to optimize the level of exposure could present barriers to the implementation of complex health and social interventions [7,8,9,10]. In relation to this, Hasson in 2010 introduced a model that systematically assesses the possible influence of the aforementioned aspects to the implementation process [7].

However, reports of process evaluation for intervention studies in the area of childhood obesity are still not common. Childhood obesity has been recognized as a serious health challenge worldwide, for which effective and feasible preventive interventions are critically needed [11,12]. If process evaluation was reported, studies normally limited it to assess aspects of fidelity, dose, and reach, while few also included the dimension of context [12,13,14]. Thus, there is a need to carry out broader process evaluations of local efforts that would provide detailed contextual descriptions that could help understand achievements or lack of results of the intervention being assessed.

The aim of the present study is to describe process evaluation measures of a school-based obesity prevention program in Lima and Callao, Peru, and to assess the factors that influenced the implementation process of the intervention leading to the mentioned process outcomes results. To achieve this, we have reported on two specific process outcomes for the various program activities (dose delivered and reach), the satisfaction of students and teachers to specific activities, and provided an overview of main factors affecting the implementation of the program.

## 2. Materials and Methods

### 2.1. Outline of the Overall Research Project—The “Health Promoting Schools” Study Design

The school-based obesity prevention program, focus of the present study, is part of an overall health promotion study titled “Health Promoting Schools” (HPS), an Official Development Assistance (ODA)-funded project by the Korea International Cooperation Agency (KOICA). The HPS intervention was designed by KOICA and Yonsei University’s Global Health Center as a quasi-experimental study targeting adolescents of six public high schools located in Lima and Callao, Peru, among which four were allocated to receive the intervention. The overall aim of the HPS intervention was to improve the mental and physical health status of adolescents by decreasing the risk factors of suicide ideation and preventing obesity. For the first, risk factors comprised depression, substance use behavior, and abuse experience, and for the latter, the targeted risk factors included unhealthy dietary behaviors and physical inactivity among the participants. The HPS intervention was implemented throughout the coordination of three teams, a design team composed of two researchers, a monitoring team, and an implementation team. Both the design and monitoring teams were based in the KOICA Peru office; the design team (composed by a Professor experienced in global health programs and a nurse experienced in school health promotion) coordinated with the different collaborating organizations at all levels about program activities, while the monitoring staff received the self-administered forms and logbooks every week, and was in continuous contact with each school implementation team regarding execution of activities, reporting and local-level planning and coordination. Local implementation teams per school consisted of one nurse and a psychologist, who were based in health centers built by KOICA and located nearby each school premises.

### 2.2. The HPS Obesity Prevention Program, Components, and Planned Delivery

The overarching concept of the HPS obesity prevention program emphasizes that by providing health education and increasing opportunities to eat healthily and stay active, students can improve their health awareness, and thereby, engage in behaviors conducive to healthy nutrition and increase of physical activity (PA), preventing the development of obesity (Table 1). The theoretical framework of the HPS obesity prevention program is based on the World Health Organization (WHO)’s HPS framework [15]. The primary domains of WHO’s HPS, on which the program components are focused, are personal health skills (students’ health education sessions); the school’s physical environment (healthy food kiosk initiative, installations of PA, and dissemination of related information); social environment (events promoting PA, Junior Health Promoters-JHP, education sessions to parents, teachers, school food kiosk staff); and school health policies (nutrition and PA-related curriculum).

The HPS obesity prevention program consisted of two main components: an education program and several environmental activities targeting the entire school community each school year. A detailed description of the elements of each component and its intended delivery per year (as stated in program plans for intervention) are presented in Table 2. In order to fit the program activities to the school context, coordination meetings were carried out between the researchers (design team) and school directors and teacher representatives during the months preceding the beginning of each school year. Program activities were implemented from April 2015 to December 2017.

The nutrition sessions of the education program for students were designed to be delivered sequentially. In addition, sessions were structured to have a media powerpoint presentation, a teamwork exercise, and a video presentation to portray a dynamic example of the subject taught during the session. Some elements of the education and environmental components varied between the first program year (2015) and consequent years (2016–2017). The Healthy Food Kiosk initiative included education sessions for kiosk staff, physical improvement by installing fruits exhibition stand and nutrition traffic lights, as well as 2-yearly evaluations.

### 2.3. Description of the Process Evaluation Model

**Figure 1:**
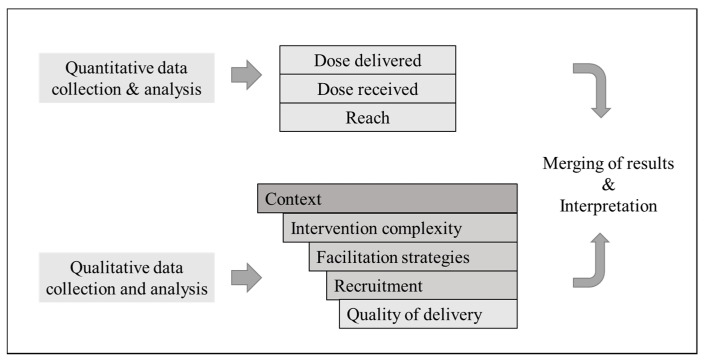
Framework of the elements for the school-based program process evaluation using mixed methods.

The HPS obesity prevention program process evaluation uses a modified model based on the work of Steckler and Linnan (2002) and Hasson (2010) (Figure 1). Based on the availability of data for the assessment of each element, our modified framework includes five elements from the framework of Steckler and Linnan, and complements it with additional factors introduced in the model developed by Hasson and referred to as moderating factors [7]. These additional moderating factors are intervention complexity (the specificity and nature of the intervention description); facilitation strategies (extent to which materials, training, and monitoring/feedback are provided to ensure uniform program delivery); and quality of delivery (the manner in which the program was delivered). An associated plan for each element included general and specific questions for data collection and analysis.

### 2.4. Data Collection and Measures

#### 2.4.1. Quantitative Data and Measurement of Process Outcomes

A self-administered form developed by the design/monitoring teams and provided to each implementation team to fill-out and report weekly was used to collect information on the actual implementation of program activities (dose delivered and reach). For the dose received, we utilized question items included in follow-up questionnaires conducted at the end of each school year for students. Quantitative process-evaluation questions of dose delivered and reach for each program component were separately defined. Examples of questions were as follows: “To what extent were all nutrition and PA sessions for students implemented?”, “Was the education program delivered to at least 70% of all students?”, To what extent were all scheduled sessions provided to parents/teachers/JHP/school food kiosk staff?, and “How many parents/teachers/JHP/kiosk staff participated in respective education sessions?”.

Dose delivered for education and environmental activities were measured using total counts of number of sessions/evaluations/materials/events carried out during the year in each recipient school, while reach was measured using total counts in terms of participants in each activity.

Dose received was assessed using satisfaction scales included at the end of the year follow-up questionnaires for students. Satisfaction to education sessions on nutrition and PA, the overall Healthy Food Kiosk initiative, the minigym or provision of selected sports equipment, and PA events were measured using a 5-point scale ranging from ‘Very Unsatisfied’ coded as 1 to ‘Very Satisfied’ coded as 5. Overall scores and respective standard deviations were calculated and expressed on a yearly basis, and the higher the score, the higher the satisfaction. In addition, satisfaction of teachers who participated in capacity-building training in 2016 and 2017 was measured in the same way as aforementioned.

#### 2.4.2. Qualitative Data and Measurement of Factors Influencing the Program Implementation

Qualitative data was collected through program-related documents and individual interviews (Table 3). Documentary data consisted of logbooks, emails, meeting minutes, and year action plans collected from December 2014 to December 2017. The logbooks, also developed by the design/monitoring teams, were designed to collect qualitative data about preparation for sessions, coordination activities, difficulties faced during implementation of activities, and persons worked with. The logbooks were filled out and reported every week. Emails and meeting minutes collected were those between the three program teams, school directors/teachers, and other related stakeholders. Program year action plans shared between program design/monitoring and implementation teams were also included. All the aforementioned documents collected were reviewed to get a better understanding about the context within which the implementation of activities took place, and to assess the characteristics of the intervention, the support procedures provided to implementation teams, the recruitment procedures established, and the style of delivery of implementers (Figure 1).

In addition, individual interviews with students, parents, teachers, implementers (nurses) and Peru officials were conducted using purposive sampling. The characteristics considered for each type of participant were as follow: students who had been enrolled during entire program duration; individual parent or both parents of a student enrolled during entire program duration who were aware of the program existence and/or participated in a program activity (preferably parents of potential student interviewees); teachers who had worked in intervention schools during entire program duration and were aware of program and/or participated in any activity; nurses who were in charge of implementing the program activities between April 2015 and December 2017; and Peru officials who had been in charge of monitoring program activities as recipient country counterpart during its entire duration.

The process to recruit the participants was established as follows: first, the Regional Health Direction in charge of supervising health-related programs in the four intervention schools was contacted via e-mail and phone by one of the authors (RCCH) to inform about the study and request permissions, given that some potential student respondents were still attending school. After receiving the respective approval, directors of the four intervention schools were separately contacted to request contact information of potential participants. Potential participants (students, parents, teachers) were later contacted by phone by nurses who implemented the program in each school who provided a general explanation of the study and inquired their willingness and consent to participate in the interview. For students still attending school, parents were provided with respective information as well. The participants who agreed to participate were later contacted by the interviewer (RCCH) to schedule date and place of the interview. Interviews were conducted in Spanish using a semi-structured interview guide covering perceptions of the program activities and their implementation. Examples of evaluation questions were how much did students/parents/teachers engage in education sessions, and how did the program characteristics, nurses, and the school/home environment influenced the implementation of education sessions and environmental activities. Interviews were carried out using broad definitions or similar examples to avoid bias towards any particular type of answer.

The Regional Health Direction recommended to include two participants per school in the case of the students, parents, and teachers’ group (24 respondents across three groups). In the case of two schools, one nurse who implemented activities during the entire program period in each was invited by e-mail and phone, while for two schools, two nurses in each were invited taking into account the resignation of the first implementer in each school (6 nurses across four schools). Finally, one Peru official who worked at the regional health direction and complied a monitoring role of the program activities during all intervention years was invited via e-mail and on-site to participate.

### 2.5. Data Analyzes

The present study has a mixed methods design, where the quantitative and qualitative data were collected and analyzed in parallel but separately, and later brought together in the interpretation of the overall results (Figure 1) [16]. The integration of both quantitative and qualitative data was done through a narrative weaving approach [16].

#### 2.5.1. Analysis of Quantitative Data

Data on dose delivered, dose received and reach from self-reported forms were first entered into an Excel workbook that was uploaded into SPSS 24.0 Statistical software to obtain descriptive statistics (frequencies, means) for each program activity per school. Dose delivered for education sessions, food kiosk evaluations and dissemination of information was expressed on a yearly basis as a percentage resulting by dividing the number of sessions/evaluations/information materials delivered in the year (e.g., 2015) by the total number of sessions/evaluations/information materials scheduled for the year and then multiplied by 100. In the case of activities concerning installation of equipment and PA events, the aforementioned formula was expressed as a percentage of activities carried out during total program duration (2015 to 2017). The reach formula established for education and environmental activities was expressed as a percentage resulting by dividing the number of participants in the activity by the total number of participants targeted and then multiplied by 100. In the case of parents’ sessions, the total number of parents per school was defined in terms of one parent per student for calculation. For walking events, information on total school administration staff and guests was not available, for which reach was expressed as a total count. Satisfaction mean scores ± standard deviations were calculated for the six selected activities as mentioned in the quantitative data section above.

#### 2.5.2. Analysis of Qualitative Data

A total of 31 individuals (23 women and 8 men) 16–56 years old, participated in the individual interviews. Interviews were held for 35–70 min, with an average interview time of 46 min. Also, RCCH took daily field-notes of the interaction with respondents and problems encountered in data collection. All interviews were recorded and transcribed verbatim, some by the first author (RCCH), and some by one Graduate student whose mother tongue was also Spanish. Transcriptions were made in Spanish and English, and RCCH listened to all interview recordings to make sure transcriptions were correct.

Qualitative data were analyzed using a deductive Qualitative Framework Analysis approach [17] using the modified model and associated plan as initial themes (Figure 1). The documentary data was analyzed together with data from individual interviews, so that themes would emerge across the two sets of data. The data analysis was carried out as follows: First, a sample of document data (34 logbooks, 30 meeting minutes, 12 action plans), and 14 interview transcripts were imported into an NVivo database (QSR International Pty Ltd., Australia) by date and read by one of the authors (RCCH) to get an overview of the content of the data collected. Second, throughout the aforementioned process, NVivo note memos were created on key issues, ideas and emergent themes expressed by each type of participant in relation to and differing from the initial themes of the tentative thematic framework. Third, RCCH extracted portions of both, the sample documentary data and data from the interview transcripts in full that corresponded to a particular issue or idea (identified as an NVivo code), after which the indexed codes were arranged deductively in charts related to the 5 elements in the process evaluation model. At this stage, an initial coding index was developed. Fourth, remaining documentary and interview transcripts data in Spanish were coded by RCCH, while EWN separately coded the interview transcripts in English. In this stage of analysis, both authors mutually compared and discussed charts in order to reach a refined coding index and thematic framework matrix [18]. Following that, the first author input preliminary descriptive accounts (summaries) of the particular issues or ideas expressed by the participants in regards to each element into a summary matrix including descriptions, sub-themes, and themes, which were finally reflected on and condensed into final themes through agreement between both authors. NVivo 12 qualitative data software was used as a tool for analysis to develop the note memos, and initial coding index. Furthermore and to check the interpretations, the initial coding index along with the documents and interview transcripts used to derive it were reviewed by one of two study researchers (Professor experienced in Global Health), who provided comments that were considered to refine the coding index and the analysis framework matrix. RCCH and EWN were versed on the HPS intervention and obesity program.

### 2.6. Ethical Approval

Ethical approval for the study was obtained from the Institutional Review Board of the Wonju Campus of Yonsei University (IRB 1041849-201909-SB-134-02). Regarding the in-depth interviews, informed consent was also obtained from each interviewee after providing information about the purpose of the study and use of the data. In the case of under-age participants, a parent present before the start of the interview signed the informed consent form instead.

## 3. Results

The results are presented regarding the modified process evaluation model (Figure 1). In order to achieve a complete insight of factors that facilitated or hampered the implementation process of the HPS obesity prevention program in Lima and Callao, Peru, we first describe the main results in terms of delivery of program components and their respective reach (Table 4 and Table 5), alongside an overview of the factors identified to have influenced the mentioned process outcomes results using illustrative quotes. Sub-themes and themes related to the elements in the process evaluation model are provided in Table 6.

### 3.1. Program Delivery: Dose Delivered of Education Sessions for Students and Other Target Groups and the Influence of Intervention Complexity, Facilitation Strategies, and Context

Between 2015–2017, the delivery of nutrition and PA sessions for students increased or was maintained across schools (78.4% average delivery of nutrition sessions planned in 2015 and 88.0% in 2017; 79.7% average delivery of PA sessions in 2015 and 93.8% in 2017). With regards to environmental activities, with the exception of parents, delivery of teachers, JHP, and school kiosk staff sessions increased over time. The education component of the HPS program (sessions for students) was considered the main activity, while sessions carried out with other target groups (parents, teachers, JHP, kiosk staff) aimed to support the healthy dietary and PA behaviors promoted among the students. Implementers mentioned about the importance given to the education component of the program, as described in action plans every year, where detailed information was available on session process and standardized PPT tools to be used. This was expressed by the quote *“KOICA had a process already defined for education sessions which was the same every year”*. Furthermore, school directors and teachers were mentioned to have had an overall key role in accommodating program sessions (especially for students) in the school curriculum for their benefit. However, teachers reported about the difficulty of schools to adopt an external program and its different activities into individual year schedules, especially during the first years, which was described as *“KOICA project activities in the first years were not easy to follow”.* The mentioned difficulty was greater for schools with both, morning and afternoon shifts (School 1 to 3), which was expressed by teachers as *“It was our first time having an external program in the school, so we needed time to adapt the KOICA activities to our everyday routine”*.

In the case of education sessions directed at parents, the complexity of being part of the overall HPS intervention influenced the delivery. Since 2016, mental health-related activities including parental sessions were increased based on the risk factors identified, thus, the obesity-related sessions targeting parents became sidelined or seen as a lower priority. As a result, only one of three scheduled sessions was implemented in 2016 (data not shown), and nutrition and PA sessions for parents were completely ruled out from the yearly plan in 2017.

Education for teachers passed from non-delivery in 2015 to full delivery of training programs carried out in both years, 2016 (not shown) and 2017. The program design team determined to outsource capacity building programs for the years 2016 and 2017 with a local University. According to a meeting minute where design team and University representatives coordinated the training program in 2016, the decision was taken *“in order to provide teachers a learning opportunity at graduate level in relation to core components of health promotion in the school setting not only for the intervention immediate impact but thinking of long-term effects for the individual, students and the school in general”*. In this way, it was expected of teachers to enroll and actively participate in training out of school working hours.

Education sessions delivered to school kiosk staff averaged 67.9% in 2015, 81.3% in 2016 (data not shown) and 100% in 2017. While in 2015, program nurses carried out the education sessions, from 2016, staff from the KOICA-built health centers located nearby school premises were requested to develop this activity. The decision was made in order for program nurses to focus on sessions for students and provide support in planning, coordination, and implementation of duties for new mental health program activities. However, nurses considered there was a lack of timely coordination and monitoring for this specific activity between design team and health center directors on occasions, which affected the implementation of some sessions (especially during 2016). This was described especially by nurses as *“I think coordination was not effective”*. In spite of this, health center directors and new implementers were perceived to make efforts to accommodate the activities into daily work agendas, though there were delays in sessions implemented.

In regards to JHP education sessions, 75% of sessions in average were carried out in 2015 and 2016 across four schools, while delivery was 100% in 2017. JHP sessions was another activity translated to health centers since 2016. In addition, in 2015, only one session was implemented in School 2, which was one of the schools that exhibited a greater difficulty to adapt some of the activities into their schedules. This was related to the fact that the school director during that year was perceived to give low importance to program activities other than school sessions. This was described by the assigned nurse as *“She (the school director) only focused on letting us carry out the students’ sessions”*.

### 3.2. Program Delivery: Dose Delivered of Other Environmental Activities

The greater focus on the mental health program since 2016, made PA events another activity that was shifted directly to schools. The quote “*We had the intention to organize the walking event and sports Olympics like KOICA did but the many activities of the school didn’t leave us time to do it*” represented the difficulty schools had to prepare and carry out both events by themselves within the school year. Thus, while both PA events were carried out in each school in 2015, there were no walking events done in 2016 across all schools, among which one (School 1) did not carry out any event until the last program year. School 1 and 2 were recognized by the nurse in charge of activities as giving a low importance to some of the program activities as compared to their own schedule, which was expressed by the quote “*The school was very attached to its normative, own plans and activities, so it was difficult to insert some of the activities into such tight schedule*”.

### 3.3. Program Reach: Education and Environmental Activities and the Influence of Recruitment Strategies and Context

All schools reached an important proportion of targeted students, with at least 75% of total students participating in all sessions per year. The average percentage of parents participating in all sessions provided across schools was 29% in both 2015 and 2016. While written notes on the school diary of students were the main method used to contact parents for activities, their participation into sessions was mainly affected by conflicts with working and personal schedules. As nurses expressed *“time availability was the biggest difficulty for parents’ sessions. We could not match the personal or working schedule of many”.* In spite of sessions that were rescheduled to be carried out in the evenings and the use of individual attendance cards, parent participation did not improve across schools in 2016. Parents themselves described their difficulty to join sessions as *“I couldn’t join sessions because of my work”* or *“I had to take care of house chores and my younger children”*.

Teachers attendance to capacity building training averaged 33.1% across schools in 2016 and 39.2% in 2017. The teachers themselves recognized extended working hours as the main barrier to their involvement in programs, since “*many worked both shifts in the intervention schools*”, while others did the same even on weekends. The program tried to adjust this by offering two schedules of the yearly training programs (3 h each on Tuesdays and Thursday evenings or 6-h sessions on Saturdays); however, attendance did not improve considerably.

Attendance of JHP to sessions increased from 47.2% in 2015, 54.7% in 2016 to 62.4% in 2017. Gathering specific students from different classrooms, school years, and shifts (morning and afternoon) for sessions during class hours was not an easy task. Thus, nurses reached an agreement with school directors to do sessions in between shifts from 2016 to avoid students losing respective class hours. However, this strategy placed a burden to JHP; those from the morning shift needed to stay after school hours while those from the afternoon shift needed to come to school early. Students described this as “*few JHP students actually participated in the sessions, maybe because the schedule was out of class hours*”. Given the aforementioned difficulty, during the second and third school year, incentives were provided for those participating in sessions, especially lunch boxes and presents like small stretching ropes and ecobags.

In regards to the extent schools’ kiosk staff participated in sessions made available, attendance in 2015 was 65% across the four schools, 57% in 2016, and 47.4% in 2017. Food kiosks worked under a year renewable contract with the school and coordination was made through directors to ensure the participation of staff in sessions. However, kiosk staff were not always willing to receive sessions, given concerns about profit. Some staff believed changes introduced to products they offered and the physical presence of uncontrolled competing vendors could affect their “business” which was described by nurses as *“they said we can offer everything healthy, but it is actually a business, if students do not want it, outside the school there are street vendors”.* To this, throughout program period, food options available outside each school were out of the influence of the program.

### 3.4. Program Delivery: Dose Received by Satisfaction of Participants with Selected Program Activities

After the first year of implementation (2015), students rated the PA events as the activity they were most satisfied with (mean score across schools 3.88), followed by the nutrition sessions from the education component (mean score 3.72). PA events were developed in full in the mentioned year across schools, while in 2017 students from School 1 considered the activity as the one they were most dissatisfied with (1.35 score) given non-implementation. At the end of the last intervention year, students were mostly satisfied with the education component of the program (3.74 mean score for nutrition sessions; 3.64 score for PA sessions), followed by the Healthy Food Kiosk Initiative with an average score of 3.63. In relation to the satisfaction of teachers who attended the capacity building training made available by the program in 2016–2017, average score across schools was above 3.9 in both years, indicating overall satisfaction with the courses (Table 5).

## 4. Discussion

The aim of the study was to describe process measures of the HPS obesity prevention program in Peru, and to assess the factors that influenced the implementation process of the intervention leading to the mentioned process outcomes results. We found that the overall delivery and reach of education sessions for students between 2015–2017 increased or was maintained across schools, a decrease in delivery of parents’ sessions, an increase in delivery of sessions for teachers, JHP and school kiosk staff, and that the delivery of PA events differed across years and schools. About reach of environmental activities, less than 30% of parents in average participated in total sessions provided yearly, while less than 40% of teachers across schools attended both capacity building training provided. In regard to dose received, the activities students were most satisfied within 2015 were the PA events and nutrition sessions, while in 2017, it was the education sessions (nutrition and PA), followed by the Healthy Food Kiosk Initiative. In addition, it was found that the complexity of the intervention the program was embedded in, the extent strategies to facilitate implementation and maintain the involvement of participants were carried out, and contextual factors across and between recipient schools were the main factors that influenced the implementation process of program activities. The findings related to intervention complexity, and facilitation/recruitment strategies coincide with different studies which argued about their potential role as barriers to implementation [7,8,9,10].

### 4.1. The Challenge of Being Part of a Wider ODA Health Promotion Intervention

In the present study, the complexity we refer to is the one that stems from being part of HPS, an ODA health-promoting school intervention that included separate mental- and obesity-related programs each with different goals and characteristics targeting the same audience and developed during the same period. In this sense, being part of a wider health promotion intervention considerably affected how the HPS obesity prevention program was planned, implemented, and evaluated. The HPS intervention worked under an agreement between the Peru Ministry of Health and KOICA Peru office, for which Peruvian stakeholders from different levels and hierarchical lines were involved from the start of the intervention. Previous studies have highlighted how the number of groups or organizational levels involved in an intervention constitute key dimensions affecting a program functioning [8,19,20,21]. In addition, the HPS obesity prevention program comprised of multiple interacting components, which increased the level of coordination between program teams and Peruvian stakeholders, making the implementation process even more complex [22].

### 4.2. The Need to Establish Better Facilitation and Recruitment Strategies

Year action plans constituted the main material provided by the design team to nurses for the implementation of activities. As the results section highlights, there was a detailed description concerning the education sessions process and tools to be used (mainly PPT slides). However, roles between design team and implementation nurses for coordination with related stakeholders (school directors, health centers) were not clearly defined, and such lack of task description became a barrier that affected the development of some activities such as PA events, JHP, and kiosk education sessions. This relates to a study were guideline implementers identified the use of multiple strategies to improve implementation success [23]. Another study that described process, impact, and outcome results of a 2-year diet and PA school program in Sweden, argued how insufficient description in action plans could help explain the low degree of implementation found across total target schools [24].

### 4.3. The Need to Assess and Monitor Contextual Influences before and during Implementation

Contextual factors greatly affected how the implementation of the school-based intervention developed. This was represented by the characteristics of each intervention school and related stakeholders which acted as barriers to the implementation of overall activities. As the mentioned section highlighted, perceived importance by school directors and teachers differed between education sessions for students and environmental components. These results coincide with extensive research that identified context as a strong impeding factor for program planning, implementation, and achievement of outcomes results in health promotion and policy research, for which arguments of a thorough assessment of local circumstances at all stages of program planning and implementation duration have been found to increase program adaptability towards the achievement of outcomes [12,13,14,25,26].

This study has different limitations. There was a lack of data collection methods for different elements that would have allowed a more comprehensive process evaluation. This included observations, questionnaires, and/or specific fidelity assessment tools. Furthermore, data availability only allowed us to describe total number of sessions/activities implemented within each year per school but not in regards to duration or other objective measures. Satisfaction scores were used to provide an overall idea of how participants responded to selected program activities. Thus, averages might represent a favoring but not strong process evaluation measure. Also, there is the possibly selective response to interviews among participants. For instance, directors and teachers who responded might have been more or less committed and positive toward the program or the program goals. In addition, we interviewed a limited range of program representatives involved in day-to-day program implementation, leaving out the probable different perceptions and needs from those in other roles.

## 5. Conclusions

The process evaluation results of the study provided an overall picture of the extent to which the different activities of the HPS obesity prevention program were carried out as planned, and showed how characteristics of the intervention including multiple interacting components, the degree insufficient task descriptions, and materials are provided to implementers, and unclear coordination lines influenced the development of activities. Our results also showed how contextual factors represented by the number of stakeholders and target groups involved, time availability and perceived importance, and commitment towards program goals impeded uniform program delivery and reach across the four target schools.

The study findings thus highlight the importance of understanding the conditions of each recipient organization (school) as well as the individual expectations and needs of the different target groups, so that planning can vary to fit activities and strategies to the context, which could subsequently ensure better program implementation.

## Figures and Tables

**Table 1:** Logic model for the Health Promoting Schools (HPS) Obesity Prevention Program.

Inputs	Materials	Immediate Impacts	Short-Term Impacts	Behavioral Impacts	Health Outcomes
Providing education sessions and carrying out environmental activities targeting students and other members of school community	Year action plans, nutrition and PA curriculum, exhibition stands, nutrition traffic lights, leaflets, banners, minigym, sports equipment, program souvenirs	Participants increased awareness of the importance of a healthy diet and the practice of PA	Development of students’ skills for consuming healthy food, abstaining from unhealthy food options and engage in more PA, parent, teacher and peer support of healthy diet and PA behaviors, food availability at school kiosk	Increased/reduced intake of healthy/unhealthy food and increased participation in PA	Prevent the development of obesity, and thus, improve the physical health status of students

Adapted from Hasson (2010). PA = physical activity.

**Table 2:** Components and Planned Delivery of the HPS Obesity Prevention Program in Lima and Callao, Peru.

Component	Years	Details	Dose
1. Education program for students	2015	Sessions on two modules–Nutrition (5 subjects) and PA (1 subject) to be delivered by implementer nurse in each intervention school throughout the school year.	1 weekly 50 min to 1.5 h session (6-week period)
	2016–2017	Reinforcement sessions on two modules–Nutrition (3 subjects) and PA (1 subject) delivered by the nurse.	1 weekly 50 min to 1.5 h session (4-week period)
2. Environmental component			
2.1 Parents’ education	2015–2016	Sessions on nutrition (2 subjects) and PA (1 subject) per school year delivered by the nurse using open discussion and teamwork.	1 monthly 45 min to 1 h sessions (3-month period)
2.2 Teachers’ education	2015	Introductory session on program, nutrition and PA subjects. Objective was to create awareness of program, specific activities in each school, and encourage support in activities and normal class hours.	1 time 2 h session
	2016–2017	Capacity-building training on health promotion, including nutrition and PA subjects designed to be delivered each year. Training was scheduled to be carried out by a local University.	1 weekly 6-h session (4-month period)
2.3 Junior Health Promoters (JHP)	2015–2017	Selection of three students per classroom per school and provision of education sessions by nurse on nutrition (3 subjects) and PA (1 subject). Aim was to support healthy behaviors and motivate the participation and interest of students in program activities.	45 min sessions
2.4 Healthy Food Kiosk Initiative (Education sessions)	2015	Sessions on 7 nutrition subjects to be delivered by implementer nurse to the staff throughout the school year	30 min sessions
	2016–2017	Sessions on 4 nutrition subjects to be delivered by health center nurse.	30 min sessions
2.5 Healthy Food Kiosk Initiative (Physical Improvement & Evaluation)	2015–2017	Physical improvement to consist of installation of 1 fruit exhibition stand and 3 nutrition traffic lights per school kiosk during program implementation span.	4 units in total
	2015–2017	2-year evaluations of kiosks to verify the availability of healthy food options, sanitation, healthy practices, infrastructure, etc. Specific formats to be used were not established.	2 times per school year
2.6 Improve school physical environment	2015	Installation of leaflets wall board units per school to exhibit leaflets including those of nutrition and PA subjects; installation of a mini gym unit per school (set of parallel bars, pull-ups bars, and abdominal benches) to promote the practice of PA during and after class hours.	2 wall board units 1 minigym unit
2.7 PA events	2015–2017	Yearly walking event following a prior coordinated route carrying banners promoting healthy nutrition and PA in the community. The event was scheduled to have the participation of the entire school community (students, parents, teachers, school administration staff) in addition to program staff and guests.	1 time 2-h event
	2015–2017	Sports Olympics to be carried out at each school during the last quarter of school year, designed to include sports competitions (volleyball, football, athletics and/or basketball or other) encouraging the wide participation of all students in each school.	1 time full-school day event
2.8 Dissemination of nutrition and PA-related information	2015–2017	Disseminate a specific number of leaflets and posters on nutrition and PA subjects developed by the implementation team per year.	600–1000 leaflets per school (2015) 500 leaflets per school (2016–2017) 50 posters per school (2015–2017)

**Table 3:** Data collection methods and data sources for education and environmental activities of the HPS Obesity Prevention Program.

Dimension	Component	Data Collection Instruments	Data Sources	Total Number Collected	Frequency of Measurement
Dose delivered	Education program	Self-administered form	Implementation Team	304	Weekly
	Environmental activities	Self-administered form	Implementation Team	228	Weekly
		Kiosk evaluation reports	Implementation Team	12	At each kiosk evaluation
Dose received	Education program	Satisfaction scales	Students	-	End of school year follow-up
		Individual interviews	Students	8	Post-intervention
	Environmental activities	Satisfaction scales	Teachers	-	After training completed
		Individual interviews	Parents, teachers	16	Post-intervention
Reach	Education program	Self-administered form	Implementation Team	304	Weekly
	Environmental activities	Self-administered form	Implementation Team	16	Weekly
Context	Both	Logbooks	Implementation team	105	Weekly
		E-mails	Design, monitoring & implementation team, school directors, teachers	248	During program implementation
		Meeting minutes	Design team, implementation team	30	At each meeting
		Individual interviews	Parents, teachers, implementation team, Peru official	23	Post-intervention
Intervention complexity	Both	Year action plans	Design team	12	Before start of school year
		Individual interviews	Teachers, implementers	14	Post-intervention
Facilitation strategies	Both	Year action plans	Design team	12	Before start of school year
		Logbooks	Implementation team	105	Weekly
		E-mails	Design, monitoring & implementation team	124	During program implementation
		Individual interviews	Implementers	6	Post-intervention
Recruitment	Both	Year action plans	Design team	12	Before start of school year
		Logbooks	Implementation team	105	Weekly
		E-mails	Design, monitoring & implementation team, school directors, teachers	124	During program implementation
		Individual interviews	Parents, teachers, implementers	22	Post- intervention
Quality of delivery	Both	Individual interviews	Students, parents, teachers	24	Post- intervention

**Table 4:** Results in terms of Program Delivered and Reach (April 2015 to December 2017).

	Results			
Program Component	School 1	School 2	School 3	School 4
Classrooms	19	18	17	10
Total students T1–T3 (n)	825–918	554–603	417–495	352–381
Education program				
Nutrition sessions ^a^ T1–T3 (n) (%) +	64–48 (67.4–84.2)	75–49 (83.3–90.7)	64–45 (75.3–88.2)	48–27 (96.0–90.0)
PA sessions ^b^ T1–T3 (n) (%) +	12–19 (63.2–100.0)	18–18 (100.0–100.0)	17–13 (100.0–76.5)	4–10 (40.0–100.0)
Reach students ++	586–823 (71.0–89.7)	458–444 (82.7–73.6)	314–368 (75.3–74.3)	319–324 (90.6–85.0)
Environmental activities				
Parental health education				
Sessions T1–T2 ^c^ (n) (%) +	3–1 (100.0–33.3)	3–1 (100.0–33.3)	3–1 (100.0–33.3)	3–1 (100.0–33.3)
Reach parents ++	206–220 (25.0–25.4)	108–127 (19.5–22.9)	103–129 (24.7–30.9)	205–210 (58.2–55.1)
Teachers’ health education				
Total teachers T1–T3 (n)	67–64	34–35	30–26	15–23
Sessions T1–T3 (n) (%) +	0–16 (0.0–100.0)	0–16 (0.0–100.0)	0–16 (0.0–100.0)	0–16 (0.0–100.0)
Reach teachers ++	0–23 (0.0–35.9)	0–10 (0.0–28.6)	0–14 (0.0–53.9)	0–11 (0.0–47.8)
Junior Health Promoters				
Total selected T1–T3 (n)	95–46	54–54	52–48	30–33
Education sessions T1–T3 (n) (%) +	4–4 (100.0–100.0)	1–4 (25.0–100.0)	4–4 (100.0–100.0)	3–4 (75.0–100.0)
Reach Junior Health Promoters ++	33–13 (34.7–28.3)	25–47 (46.3–87.0)	23–25 (44.2–52.1)	28–28 (93.3–84.9)
Healthy Food Kiosk				
Food kiosk education (Staff)	9–9	3–4	5–2	3–4
Sessions T1–T3 ^d^ (n) (%) +	7–4 (100.0–100.0)	3–4 (42.9–100.0)	6–4 (85.7–100.0)	3–4 (42.9–100.0)
Reach kiosk staff ++	6–4 (66.7–44.4)	2–2 (66.7–50.0)	3–1 (60.0–50.0)	2–2 (66.7–50.0)
Food kiosk improvement				
Total fruits exhibition stands installed (n) (%)	1 (100.0)	1 (100.0)	1 (100.0)	1 (100.0)
Total nutrition traffic lights installed (n) (%)	3 (100.0)	3 (100.0)	3 (100.0)	3 (100.0)
Food kiosk evaluation T1–T3 ^e^ (n) (%) +	1–1 (50.0–50.0)	1–1 (50.0–50.0)	1–2 (50.0–100.0)	1–1 (50.0–50.0)
Total leaflets wall board installed (n) (%)	2 (100.0)	2 (100.0)	2 (100.0)	2 (100.0)
Total minigym installed (n) (%)	* 1 (100.0)	1 (100.0)	* 1 (100.0)	1 (100.0)
Total walking events carried out T1–T3 (n) (%)	1 (33.3)	2 (66.7)	2 (66.7)	2 (66.7)
Reach total participants T1–T3 (n)	825–0	670–?	370–575	390–401
Total sports Olympics carried out T1–T3 (n) (%)	1 (33.3)	3 (100.0)	3 (100.0)	3 (100.0)
Reach students T1–T3 (n) (%) ++	384–0 (46.5–0.0)	216–? (39.0–?)	310–? (74.3–?)	120–? (34.1–?)
Leaflet distribution T1–T3 (n) (%) +	1000–500 (100.0–100.0)	700–500 (100.0–100.0)	700–500 (100.0–100.0)	600–500 (100.0–100.0)
Poster distribution T1–T3 (n) (%) +	25–50 (50.0–100.0)	25–50 (50.0–100.0)	25–50 (50.0–100.0)	25–50 (50.0–100.0)

NOTE: PA = physical activity; ? = activity took place but registration failed; * = provision of other selected sports equipment instead of mini gym. ^a^ Five nutrition sessions per classroom were planned for 2015 (T1) and three for 2016–2017 (T2–T3); ^b^ One physical activity session per classroom was planned each year 2015–2017 (T1–T3); ^c^ Three parental education sessions were planned for 2015–2016 only (T1–T2); ^d^ Seven cafeteria education sessions were planned for 2015 (T1) and four for 2016–2017 (T2–T3); ^e^ Two-year evaluations; + Formula = number of sessions delivered in the year/total sessions scheduled for the year × 100; ++ Formula = number of participants in the activity/total number of targeted participants × 100.

**Table 5:** Mean Satisfaction Scores by Students and Teachers in Relation to HPS Obesity Prevention Program Activities.

Category	School 1		School 2		School 3		School 4	
	2015	2017	2015	2017	2015	2017	2015	2017
Education Program								
Nutrition Education	3.66 ± 1.05	3.78 ± 1.01	3.86 ± 1.04	3.73 ± 1.06	3.86 ± 1.11	3.75 ± 1.06	3.51 ± 1.08	3.71 ± 0.98
PA education	3.60 ± 1.06	3.61 ± 1.08	3.53 ± 1.09	3.65 ± 1.01	3.69 ± 1.08	3.73 ± 1.12	3.60 ± 1.13	3.60 ± 1.07
Environmental Activities								
Healthy Food Kiosk Initiative	3.98 ± 1.05	3.80 ± 1.12	3.48 ± 1.19	3.70 ± 1.24	3.52 ± 1.12	3.44 ± 1.20	3.46 ± 1.06	3.60 ± 1.23
School PA environment (minigym or equipment)	3.41 ± 1.18	3.42 ± 1.28	3.42 ± 1.17	3.60 ± 1.13	3.84 ± 1.18	3.67 ± 1.19	3.40 ± 1.23	3.59 ± 1.30
PA events	3.37 ± 1.51	1.35 ± 1.23	3.96 ± 1.19	3.71 ± 1.21	4.27 ± 1.06	3.92 ± 1.22	3.93 ± 1.11	3.91 ± 1.21
Teacher education ^a^	3.98 ± 1.07	4.42 ± 1.03	3.94 ± 1.11	3.95 ± 1.07	4.06 ± 1.5	4.41 ± 1.12	4.02 ± 1.07	4.34 ± 1.03

^a^ Capacity-building training carried out in 2016 and 2017 only.

**Table 6:** Descriptions, Sub-themes and Themes Identified from the Qualitative Analysis on Factors Affecting Program Implementation.

Description	Sub-Themes	Themes
- Increasing mental health-related activities targeting students, parents, and teachers	(1) Greater prioritization of mental health program affects program activities and their implementation	(1) Complexity of the HPS intervention
- Nurse staff supported psychologists in new activities		
- Responsibility of some components was transferred directly to local stakeholders	(2) Change of responsibility increases providers and the need for coordination and component description	
- Unclear coordination strategies between program design and implementation teams on occasions	(1) Lack of program clarity for new implementers	(2) Limited strategies to facilitate program implementation
- Lack of timely coordination and monitoring of components with school and health centers.		
- Lack of guidelines provided to schools for PA events		
- Detail of education sessions was given in plans	(2) Guidelines for education sessions	
- Parent sessions and walking events were informed in advance through notices in students’ school diary	(1) Program strategies to adapt to contextual factors	(3) Difficulties to maintain the participation of subjects
- Time availability was the main difficulty for parents in attending the sessions		
- It was very difficult to gather JHP from different years, classrooms, and shifts to sessions		
- JHP needed to make an effort themselves to attend sessions		
- Some presents and refreshment sets were given to JHP assisting sessions		
- Could not control street vendor or stores outside the school selling unhealthy foods	(2) Food environment outside school affects willing of kiosk staff to receive sessions	
- Kiosk staff were less willing to receive sessions because profit concerns by changes promoted by program		
- Parents and teachers working long hours during the week and even on weekends	(1) Low participation of parents to sessions and low participation of teachers to program training	(4) Context
- Commitment of school directors and teachers representatives to adapt students sessions to school plans	(2) Commitment of school stakeholders for complying with education and environmental components	
- Environmental activities were not given the same importance by school stakeholders		
- Stakeholders from different levels, complex hierarchical lines, and organizational procedures involved in the program	(3) Multidisciplinary character of the program	
